# Chancen niederschwelliger online Peer-to-Peer Begleitung für Jugendliche am Beispiel der Online-Plattform OPEN

**DOI:** 10.1007/s00729-021-00188-z

**Published:** 2021-12-13

**Authors:** Andrea Jesser, Susanne Schmalwieser, Anna-Lena Mädge, Caroline Culen, Beate Schrank, Markus Böckle

**Affiliations:** 1 Wissenschaftliche Arbeitsgruppe, D.O.T. – Die offene Tür, Karl Landsteiner Privatuniversität für Gesundheitswissenschaften, Dr. Karl-Dorrek-Straße 30, 3500 Krems, Österreich; 2 Department für Psychotherapie und Biopsychosoziale Gesundheit, Donau-Universität Krems – Universität für Weiterbildung, Dr.-Karl-Dorrek-Straße 30, 3500 Krems an der Donau, Österreich; 3 Österreichische Liga für Kinder- und Jugendgesundheit, Gerstnerstraße 3, Hofgebäude, 1150 Wien, Österreich; 4 Klinische Abteilung für Erwachsenenpsychiatrie, Universitätsklinikum Tulln, Alter Ziegelweg 10, 3430 Tulln, Österreich

**Keywords:** COVID-19, Jugendliche, Peer-to-Peer, Online-Beratung, Psychische Gesundheit, COVID-19, Adolescents, Peer-to-peer, Online-counselling, Mental health

## Abstract

Im Jugendalter stehen Menschen vor einer Reihe an Entwicklungsaufgaben und biographischen Herausforderungen. Nicht immer fühlen sich die Jugendlichen den Problemen gewachsen, die dieser Lebensabschnitt mit sich bringt und so kommt es mitunter zu erheblichen psychischen Belastungen sowie zu Konflikten und Krisen. Die COVID-19-Pandemie hat die Situation junger Menschen zusätzlich verschärft, die mehr als andere Altersgruppen von den Auswirkungen der Lockdown-Beschränkungen betroffen sind. Dazu zählen insbesondere Einsamkeit, soziale Isolation und Zukunftsängste. Aus unterschiedlichen Gründen finden Jugendliche oft keinen oder erst spät Zugang zu psycho-sozialer Unterstützung. Niederschwellige Hilfsangebote sind derzeit rar und decken den vorhandenen Bedarf nicht ab. Wir stellen in diesem Paper die Plattform OPEN vor, über die Jugendliche online, anonym und kostenlos mit geschulten und supervidierten jugendlichen Peer-Begleiter_innen in Kontakt treten und sich über ihre Probleme austauschen können.

## Abstract (en)

During adolescence, young people face a number of developmental tasks and biographical challenges. They do not always feel able to cope with the problems of this stage of life, and as a result they sometimes experience considerable psychological stress as well as conflicts and crises. The COVID-19 pandemic has further exacerbated the situation of young people, who are more affected by the effects of lockdown restrictions than other age groups. In particular, these include loneliness, social isolation, and fears about the future. For various reasons, young people often do not find access to psycho-social support, or only at a late stage. Low-threshold support services are rare and do not meet the existing needs. In this paper, we present the platform OPEN, which allows young people to get in touch with trained and supervised peer companions online, anonymously and free of charge, and to talk about their problems.

## Jugend als Lebensphase

Die Jugend ist ein Lebensabschnitt, der ganz besondere biografische Herausforderungen und biopsychosoziale Veränderungen birgt (Ravens-Sieberer et al. 2021). Junge Menschen lösen sich zunehmend von den Eltern und der Kernfamilie ab, sie treffen wichtige Entscheidungen für ihren weiteren Lebensweg und Selbständigkeit und Eigenverantwortlichkeit werden immer wichtiger. Nicht immer fühlen sich die Jugendlichen den Herausforderungen gewachsen, die dieser Lebensabschnitt mit sich bringt und so kommt es mitunter zu erheblichen psychischen Belastungen sowie zu Konflikten und Krisen (Cicognani 2011). Unsicherheiten und Probleme in Bezug auf die eigene Identitätsfindung, zwischenmenschliche Beziehungen und Emotionsregulation sind in diesem Alter hoch prävalent (Weinstein und Selman 2016). Das Stresserleben junger Menschen ist im Vergleich zu vorhergehenden und nachfolgenden Lebensphasen besonders hoch (Lüdeke und Linderkamp 2020) und kann in verschiedenen Lebensbereichen auftreten, wie etwa Schule, Freizeit, Eltern, romantischen Beziehungen oder Zukunftsplänen (Lazarus und Folkman 2011). Aber auch psychische Erkrankungen haben ihren Ursprung im Jugendalter (Ihle und Esser 2002).

## Psychische Gesundheit bei Jugendlichen

Ein Blick auf das Spektrum der Erkrankungen im Kindes- und Jugendalter zeigt bereits seit längerem eine Verschiebung der Erkrankungen von den somatischen hin zu den psychischen Störungsbildern (Kamper 2015). Psychische Erkrankungen im Jugendalter sind in etwa gleich häufig wie im Erwachsenenalter. Eine Längsschnitterhebung unter österreichischen Schülerinnen und Schülern im Rahmen der Studie Health Behaviour in School-aged Children (HBSC) zeigt, dass die Anzahl junger Menschen mit häufigen psychischen Beschwerden seit 2010 stark gestiegen ist und diese bei 16 bis 25 % der Schüler_innen auftreten. Gereiztheit und schlechte Laune kommen am häufigsten vor, gefolgt von Einschlafproblemen, Nervosität und Niedergeschlagenheit (BMASK 2018). Die MHAT-Studie (Mental Health in Austrian Teenagers) kommt zu dem Ergebnis, dass 16,5 % der Jugendlichen zwischen 10 und 18 Jahren psychische Probleme angeben, darunter vor allem psychosomatische Beschwerden, Gedankenkreisen, sozialen Rückzug, ängstliche oder depressive Verstimmungen (Philipp et al. 2018). Häufig beschreiben die Jugendlichen mehr als einen Bereich, in dem sie Beschwerden erleben. Skalen zur Bestimmung psychischer Erkrankungen, etwa nach ICD10 oder DSMV, zeigen sogar eine Prävalenz von 23,9 %; am häufigsten sind dabei Angststörungen und Störungen der psychischen und neuronalen Entwicklung mit 9,5 % bzw. 6,5 % (Wagner et al. 2017).

## Jugendliche und Corona

Die COVID-19 Pandemie und ihre Folgen haben zu einer weiteren Verschlechterung bestehender und zur Entwicklung neuer Probleme für Jugendliche geführt. Im Frühjahr 2020, von Oktober 2020 bis Februar 2021 und ein weiteres Mal im April und Mai 2021 wurden in Österreich Schulen und Ausbildungseinrichtungen geschlossen und Ausgangsbeschränkungen für die Bevölkerung verhängt. Dadurch brachen für Jugendliche essentielle soziale Kontakte zu Mitschüler_innen, Kolleg_innen und Freund_innen ab. Tagesstrukturen veränderten sich von heute auf morgen. Die Vermittlung von Lerninhalten erfolgte mit Hilfe von Online-Plattformen, ohne dass dafür ausreichend Erfahrungen bei den Schüler_innen, Lehrlingen oder dem Lehrpersonal vorhanden gewesen wären. Stark belastete Eltern von Kindern und Jugendlichen übernahmen im Home Office zusätzliche Betreuungspflichten. Zu Ängsten um die eigene Gesundheit und die von Angehörigen und Freund_innen kamen Ängste um die eigene berufliche Zukunft und die langfristigen sozio-ökonomischen Folgen der Pandemie (Mazumder et al. 2021). Biografisch betrachtet stellen die Schließungen von Ausbildungseinrichtungen eine Unterbrechung von Ausbildungs- und Erwerbsbiographien dar und es bleibt offen, welche Spuren dies in den Lebens- und Erfahrungszusammenhängen junger Menschen hinterlässt.

Die starke Einschränkung sozialer Kontakte – vor der Pandemie traf sich ein Großteil (70 %) der jungen Menschen täglich oder mehrmals pro Woche mit Freund_innen (Feierabend et al. 2020) – wird in der Literatur als Risikofaktor für psychische Erkrankungen beschrieben. Die Interaktion mit Gleichaltrigen ist gerade für Jugendliche ein wichtiger Aspekt der biopsychosozialen Entwicklung: In komplexen Peer-Beziehungen haben Akzeptanz, Ablehnung und Anerkennung durch Gleichaltrige einen hohen Stellenwert. Die Neuorientierung an gleichaltrigen Peers erleichtert die Entwicklung junger Menschen zu eigenständigen Erwachsenen und ermöglicht ihnen, ein Gefühl sozialer Selbstidentität zu entwickeln, während sie gleichzeitig stärkere Bindungen zu ihrer Peergruppe aufbauen (Orben et al. 2020). Gute Peer-Beziehungen schützen außerdem vor psychischen Problemen bzw. helfen dabei, diese besser bewältigen zu können (Bois-Reymond 1995; Cicognani 2011; Inchley et al. 2016; Kamper 2015). Auch das subjektive Wohlbefinden ist bei denjenigen Personen größer, die weniger häufig sozialen Rückzug als Bewältigungsstrategie nutzen.

Zunehmend mehr Studien beschäftigen sich international mit den Auswirkungen von Schulschließungen und Maßnahmen des Social Distancing auf die Lebensqualität und psychische Gesundheit junger Menschen (Octavius et al. 2020; Singh et al. 2020; Stavridou et al. 2020) und konnten negative Folgen nachweisen (Duan et al. 2020; Ezpeleta et al. 2020; Orgilés et al. 2020; Patrick et al. 2020; Ravens-Sieberer et al. 2021; Saurabh und Ranjan 2020). Auch für Österreich zeigte eine aktuelle Umfrage der Donau-Universität Krems unter über 3000 der 14- bis 20-Jährigen bei 55 % der jungen Menschen eine depressive Symptomatik, bei 47 % Angstsymptome, bei 64 % Symptome einer Essstörung und bei 23 % Symptome einer Schlafstörung. Suizidale Gedanken wurden von 16 % der Jugendlichen angegeben und das subjektive Wohlbefinden nahm signifikant ab (Pieh et al. 2021). Der Vergleich mit Zahlen aus der Zeit vor der Pandemie zeigt eine deutliche Verschlechterung des psychischen Befindens.

## Prävention und Behandlung psychischer Erkrankungen

Psychische Probleme und Erkrankungen haben nicht nur gravierende Auswirkungen auf das Wohlbefinden junger Menschen, sondern auch auf ihren Bildungs- und beruflichen Erfolg (Garrido et al. 2019; Sellers et al. 2019). Kamper konstatiert eine signifikante Verminderung der Lebensqualität und weitreichende psychosoziale Folgen, wie schulische Schwierigkeiten und Schulabbruch, geringere berufliche Chancen oder Arbeitslosigkeit (Kamper 2015). Viele psychische Erkrankungen bleiben bis ins Erwachsenenalter bestehen (Ihle und Esser 2002; Patel et al. 2007) und gehen mit einer erhöhten Wahrscheinlichkeit für andere gesundheitliche Probleme einher (Patel et al. 2007). Vor diesem Hintergrund wird die Bedeutung frühzeitiger Prävention und Behandlung psychischer Erkrankungen augenscheinlich (Hölling et al. 2014; Patel et al. 2007).

Gerade das Jugendalter ist in dieser Hinsicht jedoch eine herausfordernde Lebensphase. Einerseits sind die jungen Menschen oft noch von erwachsenen Bezugspersonen abhängig und ihre Möglichkeiten, selbst Hilfe in Anspruch zu nehmen eingeschränkt. Andererseits vertrauen sie sich mit ihren Problemen zunehmend weniger den Eltern an, als vielmehr Gleichaltrigen und Freund_innen (Bois-Reymond 1995). Dadurch können Eltern und Erziehungsberechtigte mitunter nicht ausreichend einschätzen, wann ihr Kind Unterstützung brauchen würde. Untersuchungen zeigen auch, dass Eltern eine Hilfebedürftigkeit v. a. dann sehen, wenn es durch das Problemverhalten ihres Kindes zu Störungen des Umfeldes kommt (Petermann 2005). Das ist tendenziell eher bei externalisierendem Verhalten, wie Aggression oder Hyperaktivität, der Fall, während internalisierende Verhaltensmuster, wie Depression oder Angst, die mit einem stärkeren subjektiven Leiden des betroffenen Kindes einhergehen, Eltern nicht so auffallen (Petermann 2005). Bei jungen Menschen, die bereits auf eigenen Beinen stehen, sieht man wiederum, dass Arztbesuche so weit wie möglich vermieden werden und die Hürde, mit einem Arzt Kontakt aufzunehmen, für viele Jugendliche, insbesondere junge Männer, zu groß ist (Bois-Reymond 1995). Dadurch bleiben psychische Beschwerden und Störungen bei Kindern und Jugendlichen häufig unbehandelt (Hölling et al. 2014).

Dort, wo Unterstützung für psychische Belastungen gesucht wird, stehen Familien und junge Menschen vor zahlreichen Hindernissen, wie Fehldiagnosen, Stigma oder Ressourcenknappheit. Obwohl sich in den letzten Jahren die Versorgung im Bereich der psychosozialen Gesundheit verbessert hat (Felder-Puig und Teufl 2019; Kern et al. 2013), gibt es sowohl in der Diagnostik als auch in der Therapie in Österreich nach wie vor Versorgungslücken. Trotz aller Anstrengungen ist die Kinder- und Jugendpsychiatrie immer noch ein Mangelfach (Fliedl et al. 2020; Culen et al. 2019) – auf rund 328.000 Einwohner_innen (Fliedl et al. 2018; Culen et al. 2018), respektive 30.000 Jugendliche (Wagner et al. 2017) kommt ein_e Fachärzt_in für Kinder- und Jugendpsychiatrie. Auf Psychotherapie als Kassenleistung müssen Kinder und Jugendliche mehrere Monate warten (Mauritz 2019). Ein Blick auf die Häufigkeit der Inanspruchnahme professioneller Hilfseinrichtungen im Jugendalter zeigt, dass nur 10 bis 30 % aller Jugendlichen mit einer psychischen Störung in Kontakt mit professionellen Hilfseinrichtungen kommen (Petermann 2005); von diesen wiederum wird nur ein Teil adäquat behandelt (Ihle und Esser 2002). Neuere Daten der MHAT-Studie aus Österreich zeigen, dass sich die Hälfte der psychisch belasteten jugendlichen Befragten eine entsprechende Therapie wünscht, jedoch nur wenige Jugendliche den Weg zur adäquaten Therapie finden. Dies legt den Schluss nahe, dass es nach wie vor zu wenig kostenfreie, niederschwellige, wohnortnahe Angebote gibt (Wagner et al. 2017).

## Digitale Hilfs- und Beratungsformate für Jugendliche

Im Zuge des Digitalisierungsschubes im letzten Jahrzehnt ist es im Bereich der Prävention und Therapie psychischer Erkrankungen zum Ausbau von e‑mental-health Angeboten gekommen (Kaess und Plener 2020). Es werden dafür unterschiedliche Kommunikationskanäle genutzt, wie E‑Mail, Chat, Foren oder Apps. Zunehmende Bedeutung erfahren außerdem videobasierte Kommunikationsformen, die als Annäherung an Therapie- und Beratungssettings im persönlichen Kontakt verstanden werden können. Da viele Online-Dienste auch mobil genutzt werden (z. B. E‑Mail, Chat, Websites), verschmelzen Online- und Mobilkommunikation häufig (Döring und Eichenberg 2013). Beratungen und Therapien können ausschließlich online stattfinden oder mit bereits etablierten Settings verknüpfen werden, z. B. mit dem Ziel der Anbahnung einer face-to-face Beratung oder Therapie, der Nachsorge oder ergänzenden Begleitung (Kupfer und Mayer 2019). Außerdem lassen sich verschiedene Formalisierungsgrade von Online-Angeboten unterscheiden: neben professionellen Angeboten ambulanter Einrichtungen und Beratungsstellen sowie von Psychotherapeut_innen und Psycholog_innen in freier Praxis gibt es halbformalisierte Hilfe durch semiprofessionelle oder geschulte Laienhelfer_innen in moderierten Chats, sowie informelle (Selbst‑)Hilfe durch Peers und Gleichbetroffene in öffentlichen oder teilweise öffentlichen Online-Foren oder Gruppen (Kupfer und Mayer 2019).

Gerade für Jugendliche, die als „digital natives“ mit Online-Kommunikation gut vertraut sind, können e‑mental-health Angebote einen niederschwelligen Zugang zu psychosozialer Hilfe bieten und dazu beitragen, Vorurteile abzubauen und die Gefahr einer Stigmatisierung zu reduzieren (Domhardt et al. 2018). Digitale Räume sind ein zentraler Teil der Lebenswelt Jugendlicher. Sie wachsen mit einem breiten Repertoire an Mediengeräten auf: ein überwiegender Teil der Jugendlichen zwischen 12 und 19 Jahren hat ein eigenes Smartphone (93 %), zwei von drei Jugendlichen haben auch einen eigenen Laptop oder Computer (Feierabend et al. 2020). 97 % der österreichischen Jugendlichen verbringen täglich Zeit im Netz (Schipfer 2020), wobei sich insbesondere die Smartphone-Nutzung im Zuge der Pandemie nochmals intensiviert hat und rund die Hälfte der Jugendlichen täglich fünf oder mehr Stunden am Smartphone verbringen (Pieh et al. 2021). Einen großen Teil ihrer Zeit im Netz verbringen Jugendliche mit Kommunikation (Feierabend et al. 2020) und auch für ihre Freundschaften und Peer-Beziehungen spielt Online-Kommunikation eine wichtige Rolle. Die Forschung zeigt, dass Jugendliche digitale Medien zunehmend nutzen, um Kontakt zu Offline-Bekanntschaften zu festigen, zu intensivieren und um sowohl alltägliche als auch tiefgehende Konversationen zu führen (Weinstein und Selman 2016). Während der Pandemie zeigte sich bei Personen, die vermehrt digitale Kommunikationswege nutzten, um mit nahestehenden Personen, insbesondere Freund_innen, in Kontakt zu bleiben, ein deutlich höheres subjektives Wohlbefinden (Brown und Greenfield 2021).

Die Vertrautheit mit persönlicher Kommunikation über digitale Medien begünstigt die Nutzung von e‑mental-health Angeboten und kann genutzt werden, um neue, zielgruppenspezifische Angebote für Jugendliche zu schaffen, die mitunter auf diesem Weg besser erreicht werden. In Ländern wie Australien und Schweden standen e‑mental-health Angebote in der Kinder- und Jugendgesundheit bereits vor der COVID-19 Pandemie durchgehend zur Verfügung. In Österreich gewinnen sie erst seit einigen Jahren an Bedeutung (Culen et al. 2018). Mit Eltern können Beratungen, Rezepterstellung oder auch Kontrollbefundbesprechungen über Online-Kanäle zeit- und ressourcensparend stattfinden und im Bereich der psychologischen oder psychotherapeutischen Arbeit mit jungen Menschen gab es bereits vor dem Ausbruch der Pandemie web-, mail- und chat-basierte Beratungsangebote, wie auch Lösungen zu Datensicherheit und Therapievereinbarungen (Culen et al. 2018; Oswald 2018). Eine Refundierung solcher Leistungen durch die Versicherungsträger war jedoch nicht vorgesehen. Die Pandemie hat die Landschaft der e‑mental-health Angebote (nicht nur) im Bereich der psychischen Gesundheit gravierend verändert. Das Angebot sowie die Inanspruchnahme digitaler Hilfsangeboten ist stark gestiegen (Brooks et al. 2020; Humer und Probst 2020). Auf den erhöhten Bedarf auch bei Kindern und Jugendlichen haben viele Beratungseinrichtungen mit einer Aufstockung der Angebote sowie erhöhten Personalzahlen bei ehrenamtlichen und berufstätigen Mitarbeiter_innen reagiert (Jesser et al. 2021). Nicht zuletzt finanzieren Krankenkassen nun auch Psychotherapie und Beratung über Online-Medien und Telefon (ÖBVP 2021).

## OPEN – eine Online-Plattform für Peer-to-Peer Begleitung

Mit der Online-Plattform OPEN – Open Peer Encouragement Network – sollen diese Entwicklungen aufgegriffen und Jugendliche mit einem Angebot dort abgeholt werden, wo sie sich im Alltag gerne bewegen: in digitalen Räumen und unter anderen Jugendlichen. Ausgehend von der anhaltend hohen und derzeit nochmals intensivierten Inanspruchnahme digitaler Medien bei Jugendlichen und der präventiven Wirkung von sozialen Kontakten, insbesondere zu Gleichaltrigen, wurde OPEN als Online-Plattform konzipiert, über die Jugendliche „Peers“ zwischen 14 und 21 Jahren mit jugendlichen „Peer-Begleiter_innen“ zwischen 16 und 21 Jahren Kontakt aufnehmen und sich über ihre Probleme austauschen können.

Das Angebot von OPEN wurde zudem evidenzbasiert im Rahmen eines partizipativen Forschungsprojektes entwickelt, in dem auch die Zielgruppe des Angebots und wichtige Stakeholder zu Wort kamen (Schrank und Team der Forschungsgruppe D.O.T. 2018). Die Entscheidung für eine Online Peer-to-Peer-Begleitung wurde untermauert durch Ergebnisse aus qualitativen Interviews und Gruppendiskussionen mit jungen Menschen sowie mit Eltern und Fachkräften, die mit Familien, Kindern und Jugendlichen arbeiten (Mädge et al. 2020). Die Befragten sprachen sich mehrheitlich für das Online-Angebot und eine Peer-to-Peer Begleitung aus, die für die Jugendlichen einen niederschwelligen, zeitlich und räumlich flexiblen Rahmen bietet, Unterstützung zu bekommen.

Vergleichbar mit OPEN sind in Deutschland die Initiativen „Youth-Life-Line“ und „U25“, die kostenlose Mail-Beratung für junge Menschen unter 21 bzw. 25 Jahren anbieten, die von professionell ausgebildeten Peers unter fachlicher Begleitung durchgeführt wird. Eine Bestandsaufnahme bestehender Angebote Österreich zeigt, dass es ein vergleichbares Angebot noch nicht gibt. „Rat auf Draht“ und „Time 4 Friends“ sind die einzigen niederschwelligen und anonymen Beratungen, die für Kinder und Jugendliche im gesamten österreichischen Raum zugänglich sind und beworben werden. Bei „Rat auf Draht“ können sich Kinder, Jugendliche und deren Bezugspersonen telefonisch rund um die Uhr, per Online-Kontakt (ähnlich einer E‑Mail) und in zwei verschiedenen Chat-Formaten, die zeitlich begrenzt sind (Mo–Fr 18–20 Uhr), beraten lassen. Die Beratung passiert durch ein interdisziplinäres Team von erwachsenen Expert_innen, und ist demnach keine Peer-to-Peer-Beratung, wie dies bei OPEN vorgesehen ist. Einem Peer-to-Peer-Prinzip wie OPEN folgt „Time 4 Friends“, eine Initiative des Österreichischen Jugendrotkreuz, die Jugendlichen die Möglichkeit bietet, täglich zwischen 18 und 22 Uhr via WhatsApp-Chats mit eigens ausgebildeten Jugendlichen zu kommunizieren. Im Unterschied zu „Time 4 Friends“ sieht OPEN jedoch vor, dass Jugendliche bei ihrer Anfrage mit einem passenden Peer-Begleiter oder einer passenden Peer-Begleiterin (nach Themenschwerpunkten und Interessensgebieten) gematcht werden und für die Dauer des Chats, der sich auch über längere Zeit erstrecken kann, bei diesem/dieser Peer-Begleiter_in bleiben. Das erlaubt es, dass Begleitprozesse länger dauern und eine Vertrauensbeziehung zwischen den Jugendlichen aufgebaut werden kann. Außerdem finden bei OPEN die Begleitprozesse nicht über WhatsApp statt. Dies entspricht zwar dem Nutzungstrend bei Jugendlichen, der sich weg von Email- und hin zu Chat- und Instant-Messenger-Kommunikation bewegt, birgt jedoch auch Nachteile. Bei Chat-Begleitungen verläuft die Kommunikation von Peer und Peer-Begleiter_in potenziell synchron. Dies kann jedoch bei jugendlichen Peer-Begleiter_innen zu einer Überforderung führen, vor allem, wenn emotional belastende Themen besprochen werden. Im Vergleich dazu erfolgt bei einer E‑Mail-Begleitung der Austausch vorwiegend asynchron. Vom Erhalt der Anfrage dauert es durchschnittlich länger bis zu einer Antwort, als bei einem Chat. Die zusätzliche Zeit gibt den Peer-Begleiter_innen die Möglichkeit, Antworten auf eine Anfrage zu formulieren und gegebenenfalls zusätzlich Feedback von einem professionellen Team einzuholen. Es ist keine synchrone und spontane Reaktion auf Inhalte erforderlich und Überforderungspotentiale seitens der Begleiter_innen können reduziert werden. Vor diesem Hintergrund wurde OPEN als Plattform aufgebaut, die von der Optik zwar einer Chat- bzw. Messenger-Applikation entspricht, deren Kommunikationsstruktur analog zu einer E‑Mail-Begleitung aufgebaut ist. Peers werden bei Erstellen einer Nachricht darüber informiert, dass Peer-Begleiter_innen 72 h Zeit haben, um auf Anfragen und Textnachrichten zu antworten. Sie werden darum angeregt, ihr Anliegen etwas genauer zu beschreiben und in ihrer Anfrage wichtige Inhalte einzufügen.

Wie auch bei „Time 4 Friends“ und „U25“ erhalten die Peer-Begleiter_innen bei OPEN eine Schulung, die Peer-Begleiter_innen auf Themen und Herausforderungen der Begleitung vorbereitet sowie Lösungsstrategien und schriftbasierte Möglichkeiten der Unterstützung aufzeigt. Ebenso ist bei OPEN die Supervision der Begleitprozesse vorgesehen – durch ausgebildete Kinder- und Jugendlichen- Psychotherapeut_innen und perspektivisch, durch Lehrkräfte an Schulen, die eine eigene Weiterbildung für die Betreuung der Peer-Begleiter_innen absolvieren. Aus der Forschung ergibt sich weiters die Empfehlung, jugendliche Peers und Peer-Begleiter_innen zu matchen. Etwa könnte eingerichtet werden, dass die Jugendlichen bei ihrem ersten Anschreiben sehen können, welche_r Peer-Begleiter_in noch Ressourcen hat und welche Themenschwerpunkte die Peer-Begleiter_innen angegeben haben. So könnten Jugendliche sich auch mit speziellen Themen gezielt an Peer-Begleiter_innen mit eigenen Erfahrungen oder einem eigenen Interesse in diesem Bereich wenden.

Beworben wird OPEN großflächig an Schulen und Bildungseinrichtungen, in Kinderarztpraxen sowie zielgruppenspezifisch in Jugendzentren, in Facebook‑, Instagram- und Twitter-Communities, da Forschungen gezeigt haben, dass Zugangswege zu Angeboten wie OPEN abhängig sind von Teilnehmer_innencharakteristika und über Schulen mitunter andere Jugendliche angesprochen werden, als via Internet (Bauer et al. 2019). Durch unterschiedliche Rekrutierungswege sollen junge Menschen unterschiedlicher Zielgruppen erreicht werden, um möglichst vielen Jugendlichen zu ermöglichen, OPEN als Unterstützungsangebot zu nutzen, um mit Gleichaltrigen Kontakt auf- und Hilfe anzunehmen. Das Online-Angebot ermöglicht eine flexible Integration in den Alltag der Nutzer_innen, unabhängig von räumlichen und zeitlichen Beschränkungen. Die Gewährleistung von Anonymität verringert das mit herkömmlichen Unterstützungsangeboten für psychische Probleme verbundene Stigma (Domhardt et al. 2018). Die niederschwellige Peer-to-Peer Begleitung kann eine Erstentlastung bewirken oder als Erstkontakt zu professionellen Beratungs- oder Therapiesettings überleiten (Elstad 2014).
